# Individual Traits and Pain Treatment: The Case of Hypnotizability

**DOI:** 10.3389/fnins.2021.683045

**Published:** 2021-06-02

**Authors:** Enrica Laura Santarcangelo, Giancarlo Carli

**Affiliations:** ^1^Department of Translational Research and New Technologies in Medicine and Surgery, University of Pisa, Pisa, Italy; ^2^Department of Medicine, Surgery and Neuroscience, University of Siena, Siena, Italy

**Keywords:** hypnotic susceptibility, cognitive pain control, suggestions, analgesia, imagery, autonomic, immune system, cortical plasticity

## Introduction

Pharmacological, physical and cognitive treatments reduce pain by addressing all pain dimensions. Nonetheless, drugs may be ineffective, and physical activity is not always viable. In contrast, cognitive therapies have usually good outcomes, a wide range of applicability and no side effects. Their efficacy, however, is influenced by cognitive and psychophysiological traits. In this Opinion article hypnotizability is used as a model to support the view that specific psychophysiological traits and cognitive strategies can not only reduce pain, but also modulate the pain-related autonomic and immune activity, induce cortical plasticity relevant to pain control, and assist in the choice of the most appropriate treatment.

Hypnotizability, or hypnotic susceptibility, is a multidimensional trait stable through life (Piccione et al., 1989) and measured by validated scales (Elkins et al., 2015) classifying highly (highs), medium (mediums), and low hypnotizable subjects (lows). It is associated with brain morpho-functional peculiarities (Landry et al., 2017; Picerni et al., 2019) and displays correlates in the sensorimotor (Ibáñez-Marcelo et al., 2019; Santarcangelo and Scattina, 2019), cardiovascular (Jambrik et al., 2004a,b, 2005; Santarcangelo et al., 2012) and cognitive-emotional domain (Diolaiuti et al., 2019). Both highs and lows represent about 15% of the population which consists mainly of mediums (70%).

In healthy subjects the ability to control pain through suggestions for analgesia is linearly correlated with hypnotizability scores (Fidanza et al., 2017). Hypnotic treatments, however, are particularly important for patients with neuropathic and musculo-skeletal pain (Castel et al., 2007; Carli et al., 2008; Jensen et al., 2009a,b; Jensen and Patterson, 2014), which are seldom responsive to pharmacological treatments. They have been found more effective than any other psychological intervention (Jensen et al., 2020), although high hypnotizability predicts better outcomes also in patients, owing to the highs' greater high proneness to modify their bodily condition according to suggestions, and, thus, to relax (De Benedittis et al., 1994), to their peculiar imagery abilities (Ibáñez-Marcelo et al., 2019), and to their attitude to be deeply absorbed in their own mental images (Vanhaudenhuyse et al., 2019).

## Suggestions for Analgesia

The suggestions for analgesia are requests to imagine that the experienced pain is out of the body or limited to a small part of it, or that a glove prevents one to perceive any nociceptive stimulation.

They are effective on acute/procedural, post-surgery and chronic pain (Jensen and Patterson, 2014; Facco, 2016) and, as most suggestions (Green and Lynn, 2011; Santarcangelo, 2014), can be efficaciously administered in the ordinary state of consciousness, thus not necessarily following the induction of the hypnotic state (Derbyshire et al., 2009; Paoletti et al., 2010; Santarcangelo et al., 2012). In highs, suggestions-induced analgesia, which can be focused on the sensory and/or emotional dimension of pain, is not accompanied by release of endogenous opiates, but is sustained by the modulation of the activity and connectivity of the pain matrix (Faymonville et al., 2006; Casiglia et al., 2020).

Interestingly, the suggestions for analgesia have been found effective also in healthy mediums undergoing nociceptive stimulation (Fidanza et al., 2017) and in chronic pain patients independently from hypnotizability (Elkins et al., 2007; Jensen, 2011; Jensen and Patterson, 2014; Mazzola et al., 2017; Facco et al., 2018; Sandvik et al., 2020). This can be accounted for by expectation of/motivation to analgesia (Milling et al., 2005; Krystek and Kumar, 2016; Montgomery et al., 2018; Perri et al., 2020) leading to placebo responses (Benedetti, 2013) which can reduce pain and pain-related psychological symptoms in the general population (Liossi et al., 2006; Brugnoli, 2016; Wortzel and Spiegel, 2017; Rousseaux et al., 2020). Thus, suggestions may induce non opioid analgesia in highs, opioid placebo responses in lows and, probably, mixed reactions in mediums. It is particularly interesting, in this respect, that, during hypnotic sessions, oxytocin – the hormone promoting social relationships and acquiescent behavior - is released in the hypnotist and the client and that, in the latter, the lower the hypnotizability score the larger the OXT release. A further contribution to the hypnotist-client relation could be the level of intimacy which has been associated with the polymorphism of the serotonin transporter 5-HTTLPR gene. Its variant associated with greater efficiency is not significantly associated with hypnotizability but may enhance the experience of “rapport” independently from it (Katonai et al., 2017). In brief, suggested analgesia occurs in the general population, although through different mechanisms (Santarcangelo and Consoli, 2018). In addition, in contrast to “constructive imagery” (inducing sensory experiences in the absence of actual stimulations), obstructive suggestions such as analgesia and anesthesia aimed at reducing the perception of actual sensory stimulations can be experienced also by lows if they report mental images as vivid as highs do (Santarcangelo et al., 2010). Thus, in lows, imagery and placebo responses could co-operate in the response to suggestions for analgesia.

## Neurotransmitters

In the absence of explicit suggestions for analgesia, hypnotizability related differences in pain thresholds (Hilgard, 1967; Agargün et al., 1998; Santarcangelo et al., 2013; Kramer et al., 2014) and perceived pain intensity (Santarcangelo et al., 2010) have been seldom reported. Several studies, however, describe hypnotizability-related differences in genetic polymorphisms and brain neurotransmitters content which may be relevant to pain control in the presence of suggestions and/or to the choice of pain treatments. In fact:

a. highs display the variant of OPMR1 receptors (A118G, rs1799971) characterized by low sensitivity to opiates, high consumption of opioids for post-surgery and cancer pain and low placebo responsiveness more frequently than lows, with mediums displaying intermediate frequencies (Santarcangelo and Consoli, 2018). Thus, opioid treatments are not the most appropriate in highs.
b. the Fatty-Acids- Amino-Hydrolase (FAAH) C385A polymorphism (rs324420) responsible for endocannabinoids (eCBs) degradation is not significantly different between hypnotizability groups but the polymorphism frequencies indicate a trend to higher degradation efficiency from lows to highs (Presciuttini et al., 2020). We may hypothesize that small differences in the eCBs content could be amplified by the eCBs interactions with nor-adrenegic (Scavone et al., 2013) and dopaminergic pathways (Di Filippo et al., 2008). Thus, a contribution of the FAAH polymorphism to the highs' ability to control pain by suggestions for analgesia should not be excluded.
c. oxytocin (OXT), which modulates the sensory and emotional components of pain (Poisbeau et al., 2018), can contribute to the highs' suggestions induced analgesia through activation of the endogenous opioid system and by regulating the eCBs production (Russo et al., 2012). In fact, the polymorphism of the OXT receptor gene associated with high sensitivity (rs53576) is more frequent in highs than in the general population (Bryant et al., 2013).
d. brain nitric oxide (NO) promotes the release of brain dopamine and noradrenaline (Ghasemi et al., 2019), which are involved in pain control. According to post-occlusion flow mediated dilation (FMD), the endothelial NO release at peripheral level is reduced in lows and in the general population, but not in highs (Jambrik et al., 2004a,b; Jambrik et al., 2005). If confirmed at brain level, a continuous release of endothelial NO might amplify the availability of nor-adrenaline and dopamine in highs.

## Autonomic and Immune Activity

The autonomic and immune activity are strictly related to each other (Pavlov et al., 2018; Walters, 2018; Blake et al., 2019; Elkhatib and Case, 2019; Iovino et al., 2020) in that the former modulates the immune activity (Elenkov et al., 2000; Jänig, 2014; Martelli et al., 2014) and the latter can regulate the function of brain autonomic centers (Elsaafien et al., 2019).

The mechanisms controlling acute inflammation and the associated pain are quite different from those controlling chronic inflammation and chronic pain. In particular, the pro-inflammatory cytokines produced in response to an acute body lesion excite the central nervous system by the activation of vagal afferents and, after penetration through the blood brain barrier, of brain structures which, in turn, generate anti-inflammatory responses. The networks involved in the inflammatory inhibition are: (a) the parasympathetic circuit, limited to vagal afferents and efferents; (b) the parasympathetic-neuroendocrine circuit, which is responsible for the release of corticosteroids; (c) the cytokine-vagal-sympathetic circuit, involving noradrenergic pathways and adrenal epinephrine (Pavlov et al., 2018). In the latter circuit, the mechanisms inhibiting acute inflammation and pain are distinct, triggered by specific contextual/environmental stimuli in animals and by psychological interventions in humans (Bassi et al., 2018).

High hypnotizability is associated with pre-eminent parasympathetic control of heart rate during relaxation in the awake condition with respect to lows (Santarcangelo et al., 2012), with a further shift toward parasympathetic tone after hypnotic induction (De Benedittis et al., 1994), and with greater proneness to reduce sympathetic activation during suggestions of unpleasant experiences associated with instructions for relaxation and well-being (Sebastiani et al., 2007). In contrast, and at variance with cortical and somatic correlates (Santarcangelo and Consoli, 2018), the findings of hypnotizability-related reduction of sympathetic activity associated with suggestion-induced analgesia in healthy subjects are inconsistent (De Pascalis et al., 2001; Paoletti et al., 2010; Santarcangelo et al., 2013). Theoretically, however, the autonomic peculiarities of high hypnotizable individuals – parasympathetic prevalence - should be associated with a more effective immune activity. Hypnotic treatments, in fact, upregulate the expression of immune-related genes in lymphocytes (Kovács et al., 2008), reduce salivary cortisol (Thompson et al., 2011) and immunoglobulin A in surgical patients with breast cancer (Minowa and Koitabashi, 2014), regulate auto-immune disorders (Torem, 2007), human papillomavirus (Barabasz et al., 2010), and pro-inflammatory/anti-inflammatory cytokines in elders (Sari et al., 2017).

## Cortical Plasticity

An ambitious target for chronic pain treatments should be counteracting the disadvantageous cortical plasticity associated with chronic pain, consisting of alteration in the brain gray matter volume (Xiong et al., 2017; McCarberg and Peppin, 2019; Yin et al., 2020) and in long-term potentiation in the anterior cingulate cortex and insular cortex (Zhuo, 2020).

In chronic pain patients Transcranial Magnetic Stimulation (TMS) and electrical direct Transcranial Stimulation (dTCS) are efficaciously used to modulate the activity of pain-related circuits (Klein et al., 2015; Dos Santos et al., 2018; Meeker et al., 2020) together with vagal stimulation (Costa et al., 2019). Theoretically, imaginatively induced analgesia could influence cortical plasticity (Kleim and Jones, 2008) mimicking the effects of TMS and dTCS by suggestions aimed at modulating the activity of the pain matrix (Casiglia et al., 2020) and enhancing the action of descending antinociceptive pathways (Beltran Serrano et al., 2020). The highs' stronger functional equivalence between imagery and perception/action (Ibáñez-Marcelo et al., 2019) and their greater cortical excitability (Spina et al., 2020), in fact, allow them to experience pleasant situations able to buffer the activity of the pain matrix, thus promoting the cognitive re-appraisal of their pain condition. In addition, the activity of the pain matrix itself can be reduced by suggestions (Faymonville et al., 2006; Casiglia et al., 2020) and co-operate to promote long-lasting effects. Suggestive treatment of pain, in fact, induces long-lasting analgesic effects addressing all pain dimensions (Dillworth and Jensen, 2010; Jensen et al., 2014). Of note, cortical long-lasting plasticity is induced also by neutral hypnosis that is the state experienced by highs after hypnotic induction in the absence of specific suggestions (Jiang et al., 2017).

## Conclusions

The pain matrix structure, activity and connectivity (Legrain et al., 2011) are influenced by acute and chronic pain. Our opinion is that that pain experience and physiology are modulated by the physiological correlates of hypnotizability, and that hypnotic assessment may assist in the choice of the most appropriate pharmacological treatments (a); the suggestions for analgesia are effective in both wakefulness and hypnosis and can control pain in a large majority of the general population, although through different mechanisms (b); hypnotizability is an advantageous factor in the control of pain-related autonomic and immune functions (c); hypnotizability-related cortical plasticity may counteract the effects of chronic pain on the structure and function of the pain matrix (d). In conclusion, suggestions for analgesia should be considered for any pain patient and not only after unsuccessful pharmacological treatments.

## Author Contributions

The authors equally contributed to the paper and agreed on its content.

## Conflict of Interest

The authors declare that the research was conducted in the absence of any commercial or financial relationships that could be construed as a potential conflict of interest.
